# The prognostic and diagnostic value of circulating tumor cells in bladder cancer and upper tract urothelial carcinoma: a meta-analysis of 30 published studies

**DOI:** 10.18632/oncotarget.18521

**Published:** 2017-06-16

**Authors:** Zheng Zhang, Wei Fan, Qiaoling Deng, Shihui Tang, Ping Wang, Peipei Xu, June Wang, Mingxia Yu

**Affiliations:** ^1^ Department of Clinical Laboratory, Zhongnan Hospital of Wuhan University, Wuhan, Hubei, 430071, China; ^2^ Department of Pathology, Zhongnan Hospital of Wuhan University, Wuhan, Hubei, 430071, China

**Keywords:** circulating tumor cells (CTCs), bladder cancer, urothelial cancer, prognosis, meta-analysis

## Abstract

There are inconsistent conclusions in the association between circulating tumor cells (CTCs) and urothelial cancer (UC). We performed a meta-analysis to assess the prognostic and diagnostic value of CTCs in UC. We search Medline, Embase and Web of science for relevant studies. The study was set up according to the inclusion/exclusion criteria. 30 published studies with a total of 2161 urothelial cancer patients were included. Meta-analysis showed that CTC-positive was significantly associated with tumor stage (≤ II vs III, IV) (OR = 4.60, 95% CI: 2.34–9.03), histological grade (I, II vs III) (OR = 2.91, 95% CI: 1.92–4.40), metastasis (OR = 5.12, 95% CI: 3.47–7.55) and regional lymph node metastasis (OR = 2.47, 95% CI: 1.75–3.49). It was also significantly associated with poor overall survival (OS) (HR = 3.98, 95% CI: 2.20–7.21), progression/disease-free survival (PFS/DFS) (HR = 2.22, 95% CI: 1.80–2.73) and cancer-specific survival (CSS) (HR = 5.18, 95% CI: 2.21–12.13). Overall sensitivity and specificity of CTC detection assays were 0.35 (95% CI: 0.28–0.43) and 0.97 (95% CI: 0.92–0.99) respectively. In summary, our meta-analysis suggests that the presence of CTCs in the peripheral blood is an independent predictive indicator of poor outcomes for urothelial cancer patients. It can also be used as a noninvasive method for the confirmation of cancer diagnosis. More studies are required to further explore the role of this marker in clinical practice.

## INTRODUCTION

Bladder cancer (BC) is the most common malignancy of the urinary tract and the ninth most common cancer worldwide. About 95% of bladder cancers are urothelial carcinomas histologically, with rare cases of squamous cell carcinoma and adenocarcinoma [1–3]. Upper tract urothelial carcinoma (UTUC) arising from renal pelvis or ureter is uncommon, accounting for only 5–10% of all urothelial carcinomas [1–3]. Growing evidences have suggested that there are certain significant similarities between BC and UTUC [4]. Furthermore, the behavior of both diseases is identical after adjusting for tumor stage and histological grade [5]. The standard methods for diagnosis of BC and UTUC include cytologic evaluation of urine, imaging tests and cystoscopy [6]. However, the cost for a cystoscopy is considerable expensive and it is an invasive examination with risk of complications. Furthermore, there still lacks effective biomarkers for predicting the prognosis of these patients. Alternative methods which help to diagnose and monitor in real time are urgently needed.

Circulating tumor cells (CTCs) are tumor cells that originate from a primary tumor, flowing through the bloodstream and circulating throughout the body, which may contribute to hematogenous metastasis [7]. The first report on metastatic tumor cells in the peripheral blood of cancer patients was presented by Ashworth in 1869 [8]. The detection of CTCs focuses on a new method of detecting metastatic disease earlier and being less invasive than currently available conventional methods, such as clinical manifestation and radiographic evaluation. In recent decades, a variety of approaches for detecting CTCs have been developed and applied to clinical settings, including immunocytochemistry (ICC), reverse-transcriptase polymerase chain reaction (RT-PCR), flow cytometry (FCM) and the CellSearch system, which is the only approach approved by the US Food and Drug Administration (FDA) [9]. Recently, many researchers have reached the conclusion that the presence of CTCs is a poor prognostic indicator for breast, colorectal and gastric cancers [10–13]. However, it remains unclear whether this conclusion can also apply to different clinical outcomes from urothelial cancer (UC), a definition which encompasses BC and UTUC. Several studies focusing on UC have showed that CTC-positive was associated with poor prognosis, and the number of CTCs may be associated with tumor stage and therapeutic effects [14, 15]. Whereas others failed to show such association [16–18].

With the aim to further clarify the issue, we performed a meta-analysis of published literatures to quantitatively assess the association of CTC-positive with clinicopathological features and prognosis of patients with UC. A second objective was to pool together and summarize quantitatively the available evidence with regards to diagnostic accuracy of CTC detection in UC.

## RESULTS

### Identification of relevant studies

**Figure 1:**
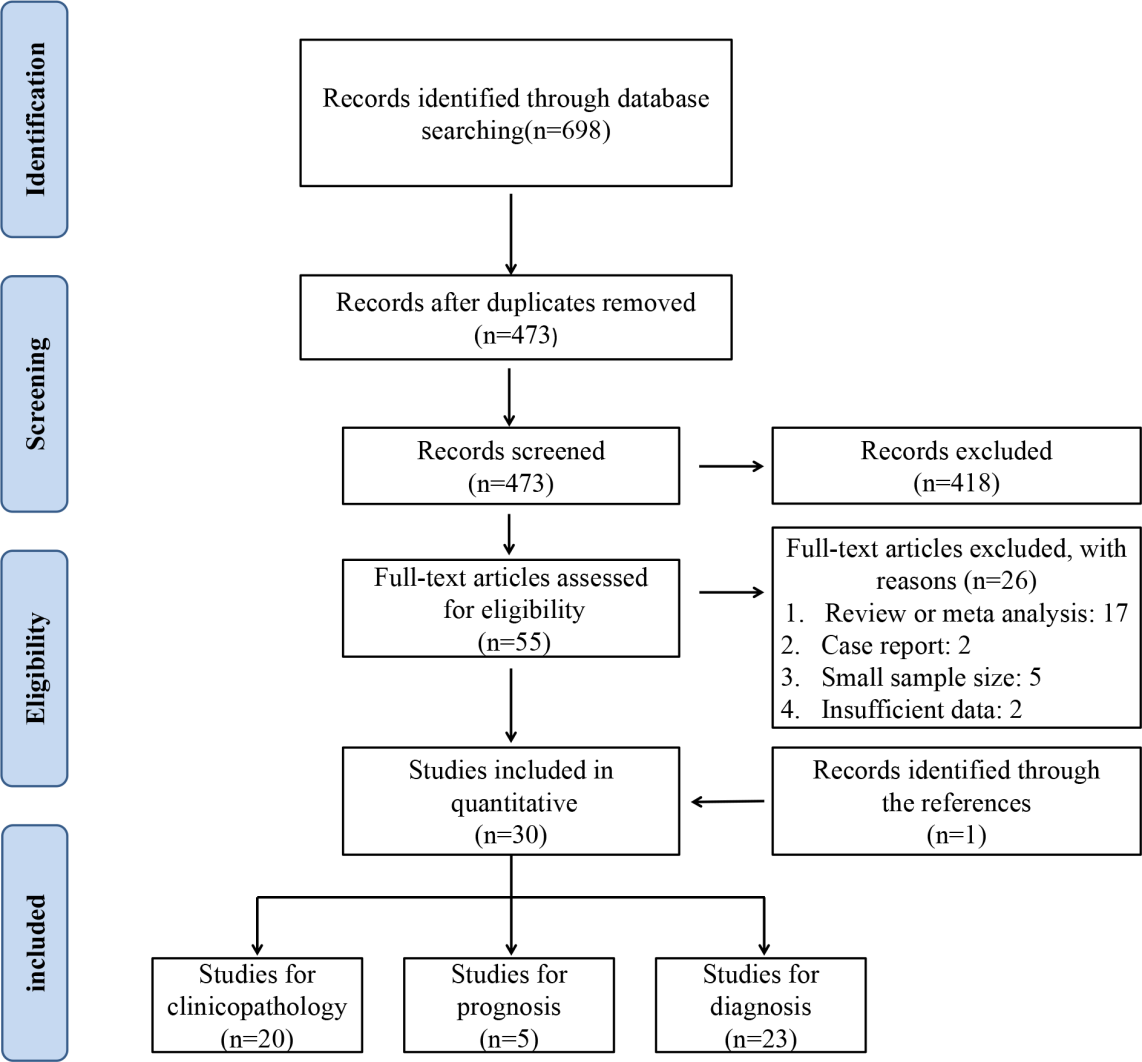
PRISMA flowchart of the selection process

A total of 698 records were identified by initial retrievement, and 473 records were selected after removing duplicates. After screening the titles and abstracts, 418 irrelevant records were excluded. There left 55 full manuscripts for detailed evaluation, of which 26 studies were further excluded for small sample size, poor study design or insufficient data. One additional study was identified by inspection of the bibliographies of previous systematic reviews [19]. Finally, 30 eligible studies were included for meta-analysis [14–43]. The flow diagram of study selection is presented in Figure 1.

### Baseline characteristics

A total of 2161 patients from 30 articles were involved. Included studies were conducted in 9 countries and published between 1999 and 2016. Between all the studies, 6 were undertaken among BC and UTUC patients, and the others only referred to BC. There were 20 studies available for the clinicopathological characteristics, 5 studies related with the prognosis and 23 studies implicated in the diagnostic accuracy of CTCs. Detection methods included CellSearch system, RT-PCR, enzyme-linked immunosorbent assay (ELISA) and other ICC. Characteristics of the included studies are shown in Table 1.

**Table 1:** Baseline characteristics of included studies

First author	Year	Country	Patients	Tumor stage (ACJJ)	Methods	Target antigen/target gene	Cut off	Prognostic outcome
Winters [23]	2015	America	BC+UTUC	II–IV	CellSearch	EpCAM	-	-
Alva [24]	2015	America	BC	II–IV	IsoFlux	EpCAM	10 CTCs/7.5 ml	-
Gazzaniga [29]	2014	Italy	BC	I	CellSearch	EpCAM	-	-
Lu [37]	2000	Japan	BC+UTUC	0a–IV	Nested RT-PCR	UPII	-	-
Retz [18]	2001	Germany	BC	0a–IV	RT-PCR	CK20	-	-
Kinjo [34]	2004	Japan	BC	0a–IV	Nested RT-PCR	MUC7	-	-
Flaig [14]	2011	America	BC	0a–IV	CellSearch	EpCAM	-	OS
Rink [42]	2011	Germany	BC	0a–IV	CellSearch	EpCAM	1 CTC/7.5 ml	OS/PFS/CSS
Li [36]	1999	America	BC	NR	RT-PCR	UPII	-	-
Naoe [15]	2007	Japan	BC+UTUC	0a–IV	CellSearch	EpCAM	2 CTCs/10 ml	-
Fujii [28]	1999	Japan	BC+UTUC	0a–IV	Nested RT-PCR	CK20	-	-
Gazzaniga [30]	2001	Italy	BC	0a–IV	RT-PCR	EGFR/UPII/CK19/CK20	-	-
Gudemann [33]	2000	Germany	BC+UTUC	0a–IV	Nested RT-PCR	CK20	-	-
Okegawa [39]	2010	Japan	BC+UTUC	I–IV	CellSearch	EpCAM	-	-
Antoniewicz [25]	2012	Poland	BC	≥ 0a	RT-PCR	EGFR/COL1A1	-	-
Ribal [17]	2006	Spain	BC	0a–IV	Nested RT-PCR	CK20	-	-
Rink [21]	2012	Germany	BC	0a–IV	CellSearch	EpCAM	-	OS/PFS/CSS
Gradilone [20]	2010	Italy	BC	I	CELLection/RT-PCR	EpCAM/Survivin	-	DFS
Gazzaniga [31]	2012	Italy	BC	0a–I	CellSearch	EpCAM	-	-
Soria [43]	2002	France	BC	0a–IV	Telomerase assay	Telomerase activity	-	-
Guzzo [16]	2012	America	BC	0a–IV	CellSearch	EpCAM	-	-
Okegawa [40]	2004	Japan	BC	0a–IV	Nested RT-PCR	UPII/CK20	-	DFS
Todenhofer [22]	2016	Germany	BC	0a–IV	RT-PCR	HER2/MUC1/EpCAM/ALDH1 TWIST/AKT2/PI3Kα	-	-
Leotsakos [35]	2014	Greece	BC	0a–IV	RT-PCR	EGFR/CK19/CK20	-	-
Osman [41]	2004	America	BC	III–IV	Nested RT-PCR	UPIa/UPIb/UPII/UPIII/EGFR	-	-
Meye [38]	2002	Germany	BC	0a–IV	ICC	CKs	-	-
Gazzaniga [32]	2005	Italy	BC	I–IV	RT-PCR	Tenascin C/EGFR	-	-
Desgrandchamps [27]	1999	UK	BC	0a–IV	ICC	CK	-	-
Allard [19]	2004	America	BC	IV	CellSearch	EpCAM	-	-
Champelovier [26]	1999	France	BC	NR	Nested RT-PCR	CK20	-	-

BC: Bladder Cancer; UTUC: Upper Tract Urothelial Carcinoma; CTCs: Circulation Tumor Cells; RT-PCR: Quantitative Reverse Transcription Polymerase Chain Reaction; OS: Overall Survival; PFS: Progression-Free Survival; DFS: Disease-Free Survival; CSS: Cancer-Specific Survival.

### Correlation of CTC-positive with clinicopathological parameters

**Figure 2:**
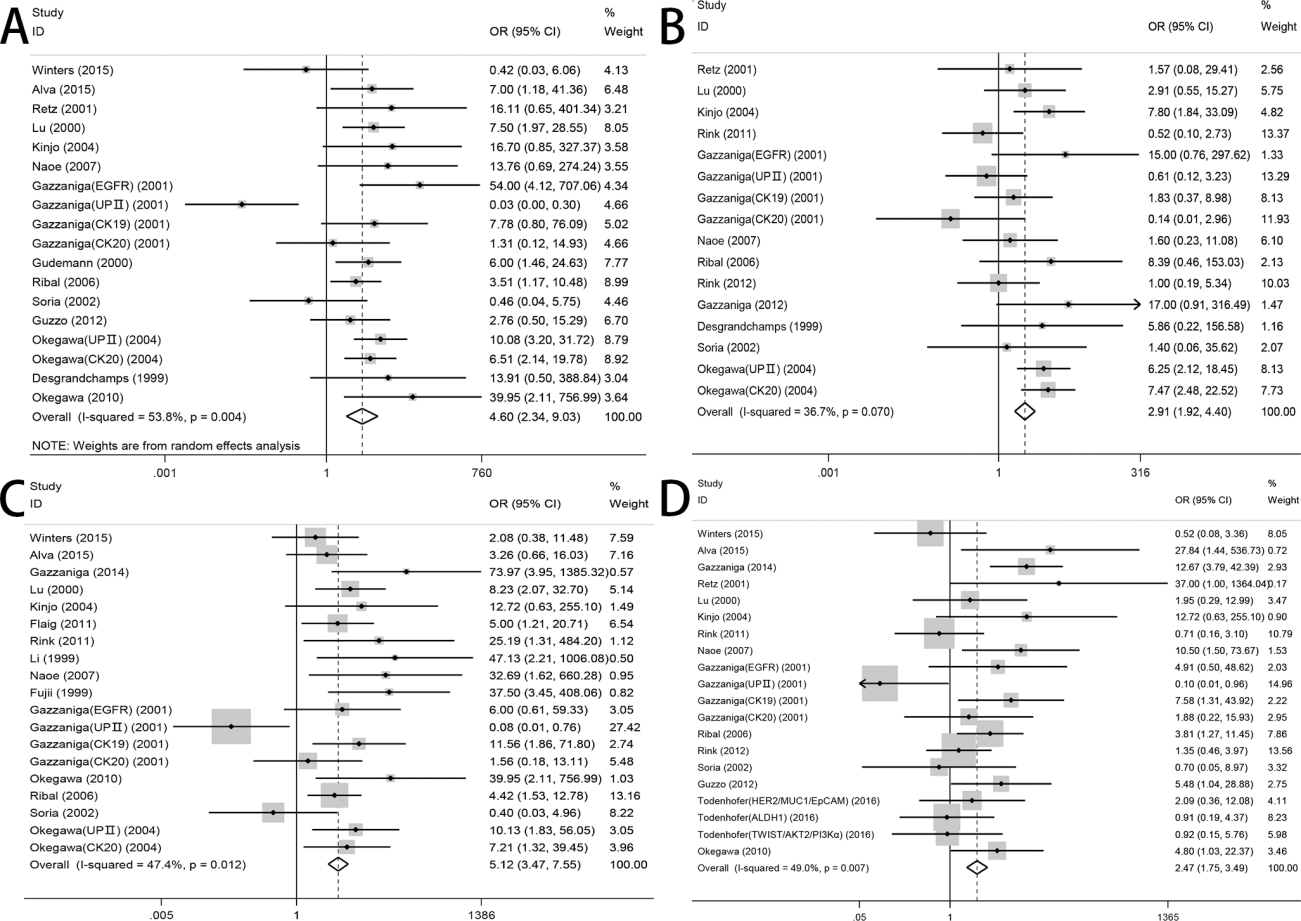
Forest plots of association between the presence of CTCs and (**A**) TNM staging, (**B**) histological grade, (**C**) disease metastasis, (**D**) regional lymph node metastasis.

**Figure 3:**
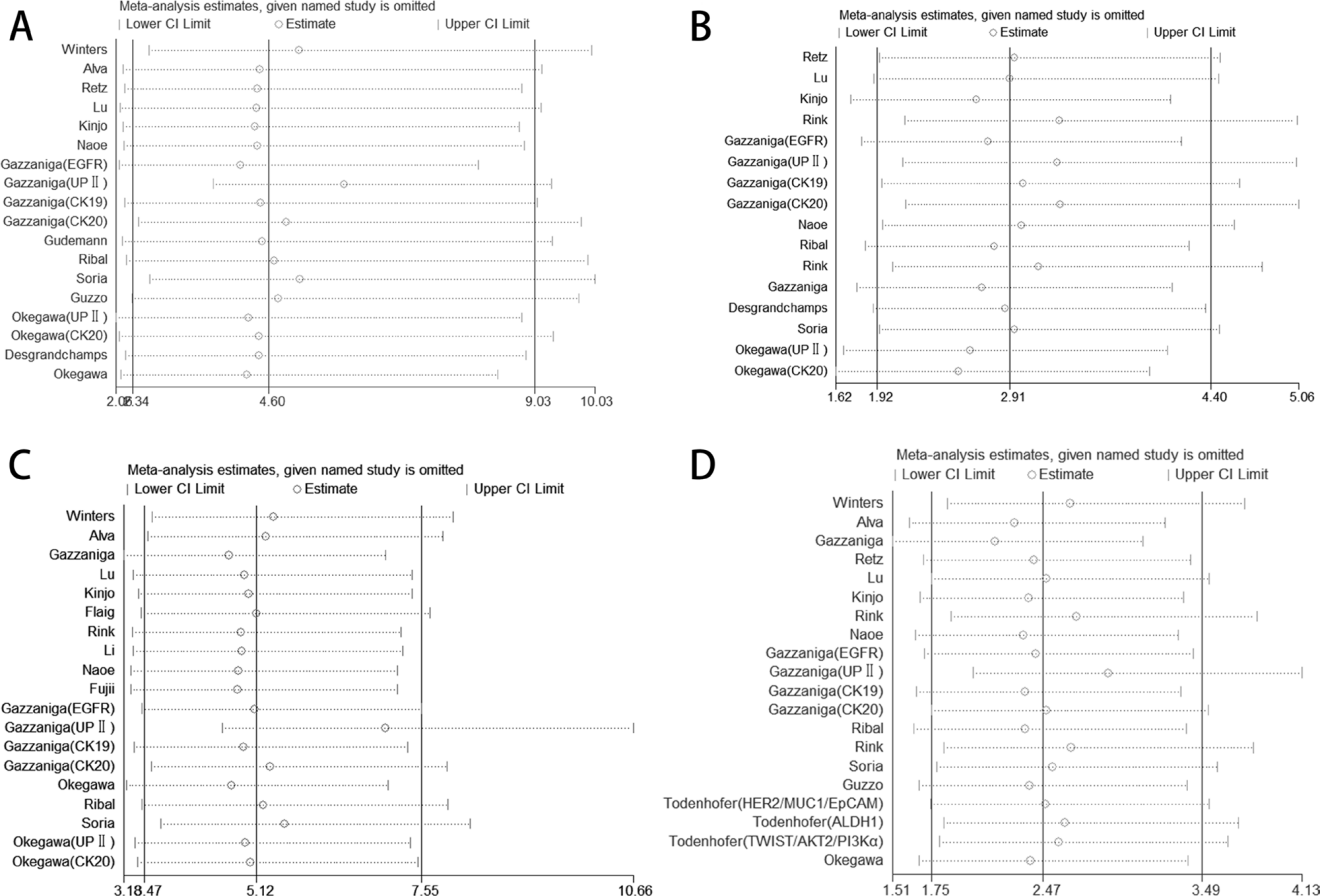
Sensitivity analysis of the studies (**A**) TNM stage, (**B**) histological grade, (**C**) disease metastasis, (**D**) regional lymph node metastasis.

We analyzed 1339 samples from 20 studies to assess whether CTC-positive was associated with UC clinicopathological parameters, including tumor stage, histological grade, metastasis and regional lymph node metastasis. The meta-analysis of all 14 relevant studies on tumor stage indicated a significantly lower incidence of CTCs in the stage ≤ II group relative to the stage III–IV group (OR = 4.60, 95% CI: 2.34–9.03; *P* < 0.001; random-effect) with moderate heterogeneity (I^2^ = 53.8%) (Figure 2A). 12 studies were used to assess the relationship between CTC-positive and histological grade. We found that CTC positivity in grade III is greater than that in grade I–II (OR = 2.91, 95% CI: 1.92–4.40; *P* < 0.001; fixed-effect) with low heterogeneity (I^2^ = 36.7%) (Figure 2B). The ORs for metastasis were available in 15 studies, and the estimated pooled OR showed a significant relationship between CTC-positive and disease metastasis: OR = 5.12 (95% CI: 3.47–7.55; *P* < 0.001; fixed-effect) with moderate heterogeneity (I^2^ = 47.4%) (Figure 2C). Results were similar for regional lymph node metastasis: OR = 2.47 (95% CI: 1.75–3.49; *P* < 0.001; fixed-effect) with moderate heterogeneity (I^2^ = 49.0%) (Figure 2D). Sensitivity analysis suggested that the results were not altered substantially by individual studies (Figure 3A–3D) except in the case of the effect of one study [30] on the combined OR of metastasis (Figure 3C). No significant publication bias was detected by Begg's test.

### Impact of CTC-positive on survival

**Figure 4:**
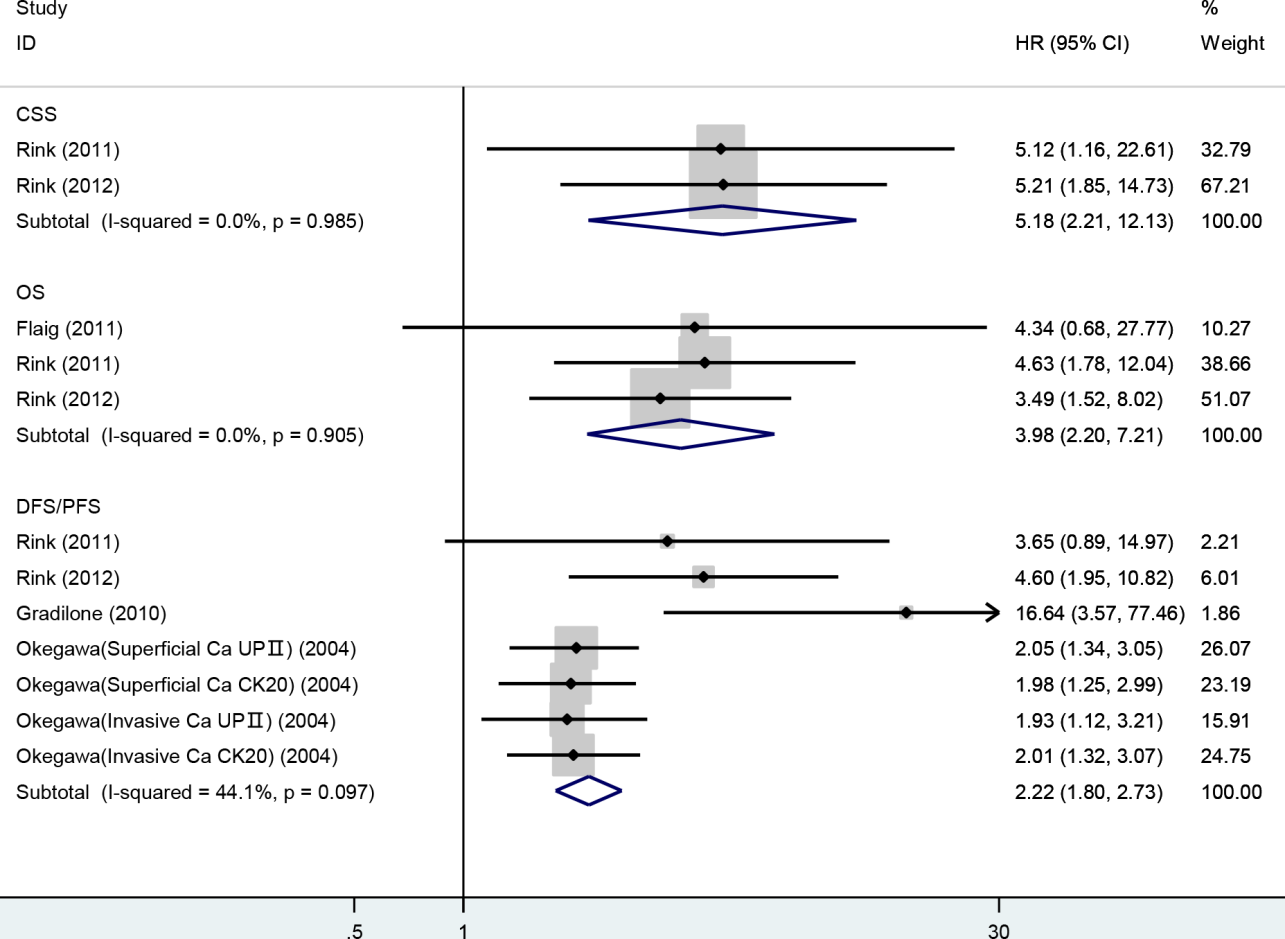
Meta-analysis of HRs for the association of the presence of CTCs with CSS, OS and DFS/PFS

Survival analysis according to CTC status was performed in 5 studies accounting for 361 patients. OS was analyzed in 3 studies. The pooled HR showed that CTC-positive was highly correlated with poorer OS and higher risk of death compared with CTC-negative: HR = 3.98 (95% CI: 2.20–7.21; *P* < 0.001). With regard to PFS/DFS, 4 studies were analyzed that comprised 317 patients. The pooled HR showed that CTC-positive was associated with a significantly increased risk of disease progression: HR = 2.22 (95% CI: 1.80–2.73; *P* < 0.001). Data on CSS were available only in 2 studies, and we found that CTC-positive was associated with a prognosis of poor CSS: HR = 5.18 (95% CI: 2.21–12.13; *P* < 0.001). No significant heterogeneity was detected in any analysis (I^2^ < 50%) (Figure 4). Sensitivity analysis suggested that no individual studies significantly affected the pooled HRs.

### Diagnostic accuracy of CTC detection

**Figure 5:**
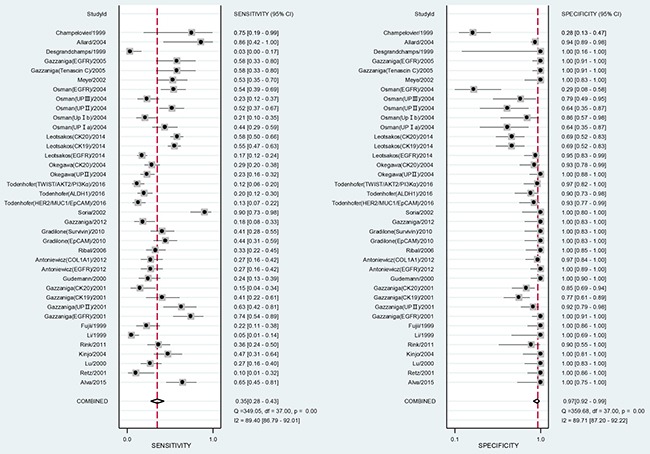
Forest plot showing study-specific (right-axis) and mean sensitivity and specificity with corresponding heterogeneity statistics

**Figure 6:**
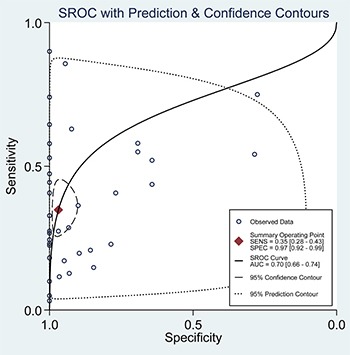
Summary ROC curve with confidence and prediction regions around mean operating sensitivity and specificity point

**Figure 7:**
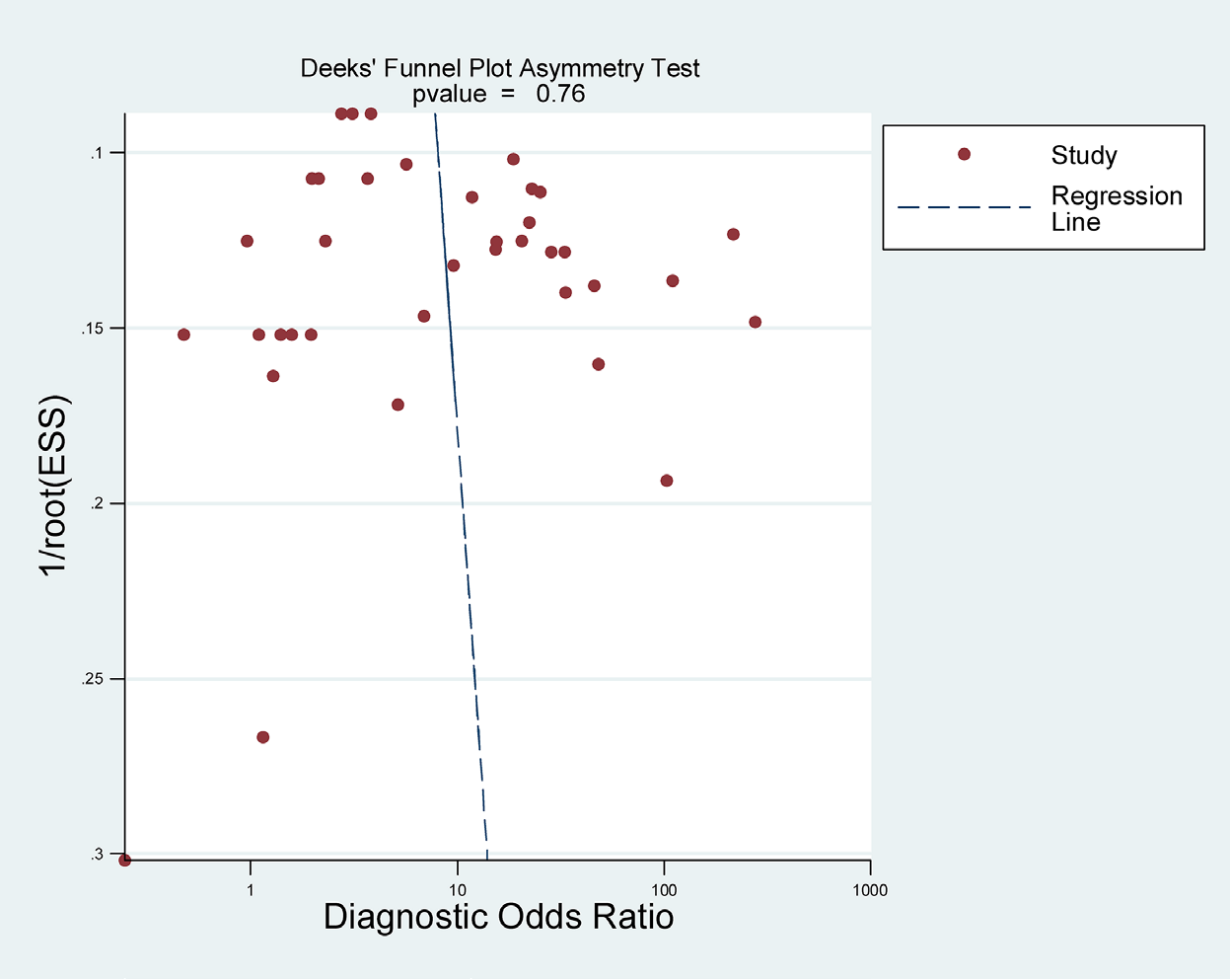
Deeks’ funnel plot with regression line

When all eligible studies and assays were pooled into the diagnostic accuracy meta-analysis, the overall sensitivity and specificity were 0.35 (95% CI: 0.28–0.43) and 0.97 (95% CI: 0.92–0.99) respectively with significant heterogeneity (I^2^ = 89.40% and 89.71%) (Figure 5). Additionally, the pooled Positive Likelihood Ratio (PLR) and Negative Likelihood Ratio (NLR) were 11.2 (95% CI: 4.5–27.5) and 0.67 (95% CI: 0.60–0.76) respectively. The diagnostic odds ratio (DOR) was 17 (95% CI: 6–43). Figure 6 presented the summary receiver operator characteristic (sROC) curve for the included studies, which presents a global summary of test performance. CTCs yielded an area under the curve (AUC) of 0.70 (95% CI: 0.66–0.74), indicating a moderate accuracy of the diagnostic test. According to the Deek's funnel plot asymmetry test, the *P* value was 0.76 for the slope coefficient, which showed there was no significant publication bias (Figure 7).

To explore the potential source of heterogeneity, we conducted subgroup analysis stratified by geographical location, control type, sample size and method. The pooled sensitivity, specificity, PLR, NLR, and DOR for each subgroup are listed in Table 2.

**Table 2:** Subgroup analysis of diagnostic accuracy of CTCs

Variables	SEN (95% CI)	SEP (95% CI)	PLR (95% CI)	NLR (95% CI)	DOR (95% CI)	AUC
Overall	0.34 (0.27, 0.42)I^2^ = 89.19	0.97 (0.93, 0.99)I^2^ = 89.71	11.8 (4.7, 29.5)I^2^ = 72.39	0.68 (0.60, 0.76)I^2^ = 78.58	17 (7, 46)I^2^ = 100	0.70 (0.66, 0.74)
Geographical location						
American	0.38 (0.21, 0.59)I^2^ = 89.32	0.85 (0.62, 0.95) I^2^ = 86.68	2.5 (0.9, 7.0)I^2^ = 80.05	0.73 (0.52, 1.02)I^2^ = 79.85	3 (1, 13)I^2^ = 99.98	0.65 (0.61, 0.69)
European	0.35 (0.26, 0.46)I^2^ = 91.08	0.98 (0.92, 0.99)I^2^ = 91.59	14.5 (4.4, 47.5)I^2^ = 61.66	0.66 (0.57, 0.78)I^2^ = 82.16	22 (6, 76)I^2^ = 99.04	0.74 (0.70, 0.78)
Asian	0.28 (0.22, 0.35)I^2^ = 55.31	0.99 (0.75, 1.00)I^2^ = 38.37	33.6 (0.9, 1307.1)I^2^ = 0	0.72 (0.66, 0.79)I^2^ = 35.06	46 (1, 1870)I^2^ = 91.58	0.46 (0.42, 0.50)
Control type						
Healthy	0.41 (0.29, 0.53)I^2^ = 90.20	0.99 (0.94, 1.00)I^2^ = 93.32	44.3 (6.8, 290.7)I^2^ = 69.57	0.60 (0.49, 0.73)I^2^ = 84.09	74 (11, 492)I^2^ = 99.71	0.79 (0.76, 0.83)
Mixed	0.30 (0.21, 0.39)I^2^ = 86.49	0.93 (0.84, 0.97) I^2^ = 83.34	4.0 (1.8, 9.0)I^2^ = 64.04	0.76 (0.67, 0.87)I^2^ = 69.49	5 (2, 13)I^2^ = 99.87	0.61 (0.57, 0.66)
Method						
Immunology-based assay	0.48 (0.23, 0.74)I^2^ = 90.19	0.98 (0.91, 0.99)I^2^ = 0	20.6 (5.2, 81.8)I^2^ = 0	0.54 (0.31, 0.91)I^2^ = 84.44	38 (8, 192)I^2^ = 99.12	0.97 (0.95, 0.98)
PCR-based assay	0.32 (0.26, 0.40)I^2^ = 89.32	0.96 (0.90, 0.98)I^2^ = 90.07	7.5 (3.1, 18.0)I^2^ = 64.81	0.71 (0.64, 0.78)I^2^ = 72.47	11 (4, 27)I^2^ = 99.93	0.61 (0.57, 0.65)
Sample size						
< 100	0.37 (0.28, 0.46)I^2^ = 85.07	0.99 (0.94, 1.00)I^2^ = 91.99	27.5 (5.6, 134.3)I^2^ = 77.31	0.64 (0.56, 0.74)I^2^ = 76.34	43 (8, 217)I^2^ = 100	0.70 (0.66, 0.74)
≥ 100	0.27 (0.18, 0.38)I^2^ = 95.20	0.93 (0.85, 0.97)I^2^ = 81.49	3.7 (2.2, 6.1)I^2^ = 53.76	0.79 (0.71, 0.88)I^2^ = 75.61	5 (3, 8)I^2^ = 99.87	0.68 (0.63, 0.72)

SEN: Sensitivity; SEP: Specificity; PLR: Positive Likelihood Ratio; NLR: Negative Likelihood Ratio; DOR: Diagnostic Odds Ratio; AUC: Area Under the sROC Curve.

## DISCUSSION

CTCs are tumor cells with specific biomarkers circulating in the peripheral blood, which can be detected in blood samples from most patients with solid tumors but rarely from healthy individuals. CTCs may have several valuable roles in monitoring disease progress and predicting treatment response of patients with malignant tumors [44]. From a clinical perspective, disease assessment by CTCs detection in the peripheral blood, with the merit of time- and cost-saving, appears acceptable to patients, and may be readily repeated as a monitoring tool. To date, encouraging results concerning the association between CTC-positive and clinical outcomes in patients with breast, prostate, and colorectal cancer have been recently published [45–47]. However, there are currently few studies on the clinical relevance of CTC-positive with UC that have included data synthesis. The present study is the first meta-analysis to systematically evaluate the associations between CTC markers and clinicopathological parameters as well as prognosis in UC patients.

The results of our study showed that the CTC-positive in peripheral blood was correlated with tumor stage, histological grade, metastasis and regional lymph node metastasis. This phenomenon indicates CTCs are more easily detected in more advanced stages (TNM) of cancer. Therefore, it is reasonable to correlate these clinicopathological characteristics with the risk of migration of malignant cells, shed from the primary tumor to peripheral circulation, which is probably an important source of metastasis directly relating to a worse prognosis. Our subsequent analysis which indicated that patients in the CTC-positive group showed poorer PFS/DFS, CSS and OS than those in the CTC-negative group was consistent with this hypothesis. From this perspective, taking the results from the present meta-analysis as references, CTCs may serve as a connecting element that bridges certain clinicopathological characteristics with progress-related and recurrence-related outcomes. The “seed and soil” theory may provide an explanation for the relationship between CTC and metastasis: tumor cells enter the blood circulation after detaching from the primary tumor and can migrate to reach distant organs, where they can implant themselves and give rise to metastasis [48]. However, additional studies with larger sample sizes and more comprehensive data about the CTCs and survival are needed to confirm this hypothesis. We also evaluated the diagnostic value of CTC in UC. However, because of several methodological limitations, the diagnostic accuracy values showed significant heterogeneity. Similar with a previous study [49], our results suggested that CTC detection assays in UC have relatively low sensitivity but high specificity. The sROC curve showed that there was great difference in the sensitivity, suggesting that improvements in the clinical and laboratory methods of detecting CTCs are required. In the subgroup analyses, it is of note that Immunology-based methods yielded higher overall sensitivity and specificity with relatively lower heterogeneity, we therefore recommend implementation of these approaches for CTCs detection in UC. As a result, CTCs detection in UC may currently have limited value as a first-line screening or diagnostic test, but may be used as a noninvasive method for the confirmation of a cancer diagnosis. During the past decades, a large majority of efforts in bladder cancer biomarker discovery and validation have been focused upon analysis of urine. Several systematic reviews [50, 51] also evaluated the significance of urinary biomarkers in bladder cancer, like quantitative nuclear matrix protein 22 (NMP22) and qualitative bladder tumor antigen (BTA). Although urinary biomarkers have its advantage of intimate contact with the primary tumor and the non-invasive nature, they may not be applicable to detection of micrometastatic as well as patients with extravesical tumors.

Some limitations of this meta-analysis need to be acknowledged. Firstly, our meta-analysis was based on data from trials whose results had been published, and we did not obtain updated individual patient data. Secondly, significant heterogeneity was found when evaluating the diagnostic value of CTCs. Although subgroup analysis was performed, the results could not fully explain the observed heterogeneity. Thirdly, CTCs detection methods were different among included studies, which might partly influence the combined results. Finally, the number of CTCs might change after treatment or surgeries [14, 23], the time of sample collection might therefore affect the detection results.

In conclusion, this is the first meta-analysis to elaborate the value of CTC in UC patients. The current evidence suggests that CTC-positive is associated with poor prognosis and clinicopathological characteristics for such patients. Therefore, it could be incorporated into risk stratification algorithms and thus aid patient management. In addition, CTCs detection may not be currently used as initial screening test but a method for confirming UC diagnosis due to the limited diagnostic sensitivity and high overall specificity. With improvements in clinical and laboratory techniques, the detection of CTCs at different time points in the future may allow real-time surveillance of dynamic changes of disease and crucially enhance our understanding of the metastatic cascade, thus facilitating novel targeted therapy approaches. However, more well-designed, high-quality and large-scale prospective studies especially about the CTCs and survival are required to further strengthen our observations and shed more light on the potential of this promising biomarker.

## MATERIALS AND METHODS

Methods of the analysis and inclusion criteria were based on the Preferred Reporting Items for Systematic Reviews and Meta-Analyses (PRISMA) statement [52].

### Data sources and search strategy

We systematically retrieved literatures up to August 2016 from the online databases Medline, Embase and Web of science without time and region restrictions. The retrievement strategy included the following keywords and MeSH terms: “Urinary Bladder Neoplasms”, “urothelial cancer”, “Urothelial carcinoma of the bladder”, “UCB”, “Bladder Cancer”, “circulating tumor cell” and “CTC”. The language was limited to English (The Medline search strategy are provided in Supplementary Appendix 1). Other relevant articles were sought by a manual search of the bibliographies of retrieved articles and review articles.

### Inclusion and exclusion criteria

Studies included in the meta-analysis had to meet all the following criterias: (1) case-control or cohort studies that assessed the association of CTC-positive with UC (bladder cancer and upper tract urothelial carcinoma); (2) ≥ 20 patients or ≥ 30 patients and controls were enrolled in each study; (3) samples used in these studies were peripheral blood.

The major reasons for exclusion of studies were (1) reviews, letters, conference abstracts or case reports and (2) articles with insufficient data or duplicated data.

### Selection of studies

Two reviewers independently screened the titles and abstracts of all records retrieved by the searches and identified studies that were potentially eligible for inclusion. Full text versions were obtained for all potentially eligible studies and these were independently assessed for eligibility by two reviewers according to inclusion and exclusion criteria. Two investigators reviewed all included articles and independently extracted data from eligible studies. Disagreements were resolved by discussion and consensus.

### Data extraction

We recorded the following information from each eligible paper: name of first author, year of publication, country, methods for detecting CTCs, cutoff value, numbers of subjects in different clinical and pathological parameters, numbers of subjects found to be positive or negative for CTC and prognostic outcomes of interest. When more than one marker was used to detect CTCs, we recorded all of these results as independent data sets. In cases where multiple blood samples were collected, we only investigated baseline (preoperative or pretreatment) value of CTC in the analysis.

### Statistical analyses

Statistical analyses were performed with Stata software (version 12.0, College Station, TX). ORs with 95% confidence intervals were used to estimate the association between CTC-positive and clinicopathological characteristics. A *P*-value less than 0.05 was considered to be statistically significant. To statistically evaluate the prognostic effect of CTC, we extracted HRs and 95% CIs on OS, PFS/DFS or CSS from multivariable analysis. If these statistical variables were not explicitly provided in the original studies, we calculated the necessary statistics on the basis of available reported data with Excels tools developed by Tierney et al. [53]. By convention, an observed HR > 1 implied a worse prognosis in the CTC-positive group in comparison to negative group. Pooled analysis of the diagnostic accuracy of CTC was also conducted.

Heterogeneity among studies was checked with the Chi-square based on Q statistical test and I^2^. Where *P* ≤ 0.1 or I^2^ > 50% indicated significant heterogeneity among studies, a random-effects model was used. Otherwise, a fixed-effects model was adopted. In order to evaluate the influence of single studies on the pooled results, we performed a sensitivity analysis using the leave-one-out approach. In addition, publication bias was evaluated by Begg's rank correlation or Deek's funnel plot asymmetry test [54].

## SUPPLEMENTARY MATERIALS FIGURES AND TABLES


