# Supplementary material for: The prognostic and diagnostic value of circulating tumor cells in bladder cancer and upper tract urothelial carcinoma: a meta-analysis of 30 published studies
Source: Oncotarget. 2017 Jun 16;8(35):59527–38. doi: 10.18632/oncotarget.18521 (PMC5601752; doi:10.18632/oncotarget.18521)
Supplement: Supplementary file 1 [file oncotarget-08-59527-s001.pdf]

The prognostic and diagnostic value of circulating tumor cells in bladder cancer and upper tract urothelial carcinoma: a meta-analysis of 30 published studies

Supplementary Materials

Supplementary Appendix 1: Medline search strategy

#1. “Urinary Bladder Neoplasms"[Mesh]
#2. urothelial cancer
#3. urothelial carcinoma of the bladder
#4. UCB
#5. bladder cancer
#6. #1 OR #2 OR #3 OR #4 OR #5
#7. circulating tumor cell
#8. ctc
#9. #7 OR #8
#10. #6 AND #9 AND English[lang]
